# Medical malpractice, defensive medicine and role of the “media” in Italy

**DOI:** 10.1186/s40248-015-0006-3

**Published:** 2015-03-26

**Authors:** Domenico M Toraldo, Ughetta Vergari, Marta Toraldo

**Affiliations:** Department of Rehabilitation, Respiratory Care Unit, San Cesario di Lecce, Local Health Unit, via Croce di Lecce, Lecce, Italy; Center for Bioethics and Human Rights, University of the Salento, Lecce, Italy; Department of Humanities, Political Philosophy, Degree course in Political Science and International Relations, University of the Salento, Palazzo Parlangeli - Via Stampacchia, Lecce, 73100 Italy; Department of Philophy, University of the Salento, Via A.C. Casetti n. 2, Lecce, 73100 Italy

**Keywords:** Clinical ethics, Defensive medicine, Malpractice, Medical error

## Abstract

**Background:**

For many years until now, Italy has been subjected to an inconsistent and contradictory media campaign. On one hand the “media” present us with bold and reassuring messages about the progress of medical science; on the other hand they are prone to kneejerk criticism every time medical treatment does not have the desired effect, routinely describing such cases as glaring examples *of* “malasanità*”,* an Italian word of recent coinage used to denote medical malpractice. Newspaper reports of legal proceedings involving health treatment are frequently full of errors and lack any scientific basis.

**Data sources:**

The published data confirm the unsustainably high number of lawsuits against doctors and medical structures, accompanied by demands for compensation arising from true or alleged medical errors or mistakes blamed on the work of health structures.

**Conclusions and implications:**

Currently Italian citizens have a greater awareness of their right to health than in the past, and patients’ expectations have risen. A discrepancy is emerging between the current state of medical science and the capacities of individual doctors and health structures. Lastly, there is a need for greater monitoring of the quality of health care services and a greater emphasis on health risk prevention.

## Review

### Introduction

Research was conducted into the most frequent causes of “malasanità” (medical malpractice) and/or “malpractice” in Italy, seeking to understand the reasons for this complex phenomenon and to provide a response to the many open questions affecting the Italian health service. Our research is based on the consultation of specialised journals and scientific periodicals, institutional, medical and scientific websites, websites of patients and citizens associations and documents published by Italian Medical Associations and insurance companies. The aim of this research is to quantify the problem and propose a series of evaluations and recommendations designed to promote the improvement of health care in Italy. Studying the phenomenon, means understanding the causes and the social and economic imbalances that characterize the provision of health care in Italy.

In Italy healthcare is one of the main sectors in which the national government and regional administrations have been applying spending reviews in order to improve public finances. The big financial deficits that have been accumulated over time and the absence of good governance in this sector, due to political and administrative incompetence, have brought about a fall in the quality of the provided health services and an unequality of them from one region to another. Because of the chronic financial problems, many citizens, especially in the South of Italy, do not have access to many types of diagnostic and therapeutic procedures that are now considered indispensable for the treatment of many diseases. Thus, the less well-to-do run the risk of being deprived of their fundamental right to health as enshrined in article 32 of the Italian Constitution [1].

### Information sources

Given the paucity of scientific literature on the subject, most of the information presented here comes from Italian institutional documents. One reason for the lack of scientific studies could be that, in recent years, this complex problem has mainly been tackled and discussed in the media rather than in suitable scientific contexts. An increasingly hostile tone has been adopted by journalists, with significant effects on public opinion, but without any corresponding development of medical services that might resolve the complex social and cultural issues being raised.

According to the *Patient Safety in American Hospitals Study*, from 2004 to 2006 healthcare mistakes caused 238,337 avoidable deaths and generated costs of 8.8 billion dollars. The study, which looked at 41 million patients in the *Medicare* public health system, was published by *Health Grades, guiding Americans to their best health*, a health rating agency. It found that patients treated in hospitals rated as high quality are 43% less likely to be affected by medical errors than patients admitted to structures considered to be of lower quality. Overall, medical errors affected almost 3% of all Medicare patients, accounting for about 1.1 million incidents due to errors occurring during the three years considered [2,3].

Unlike the United States of America, in Italy no quantitative studies of medical errors have been conducted by independent scientific research bodies. Cases have been reported by patients’ rights groups such as TDM (*Tribunale dei diritti del malato-Cittadinanza Attiva*) and private research bodies, which show the patients’ point of view and describe their complaints and accusations, but none of them is objective nor independent.

One interesting aspect is the description of “adverse events”, understood as harm arising from healthcare management rather than from the disease itself. An epidemiological study [4] analysed this phenomenon in five large Italian hospitals based on a review of 7,573 clinical cases selected from a sample of 9,000. The professionals involved in the study included doctors, pharmacists, biologists and nurses. The rate of adverse events was 5.5%, consistent with the expectations set out in the study protocol, slightly lower than the median rate of international studies (9.2%). A large proportion of these events are correlated with surgery or the use of medications. It should be pointed out that the data obtained in this study cannot be used to draw conclusions about professional responsibility, since the perspective of the research was not medical-legal but rather the quality and safety of treatment. Its usefulness lies in the support it provides for predicting adverse events, with a consequent improvement in healthcare.

In Italy the Chief prosecutor of Rome recorded a 40% increase in the number of complaints filed against doctors for alleged professional malpractice from 1999 to 2007 [5]. To organise the highly complicated casework arising from these complaints, the Prosecutor's office in Rome has set up a special work committee called the “professional fault group”.

A study commissioned by the patients’ rights group TDM [6] (19^th^ March 2007) shows that the branch of medicine most affected by complaints is Orthopaedics and Traumatology (accounting for 18% of cases), followed by Oncology (13.1%), General Surgery (12.5%), Obstetrics and Gynaecology (13%), Ophthalmology (7.1%), Dentistry (6.1%), Angiology (4.6%), Urology (3.9%), and General Medicine (2.9%). According to data from the Italian National Association of Insurance Companies (ANIA), there are 7,500 lawsuits against health structures and 8,500 against individual doctors, accounting for a total of 16,000 a year [7]. According to the National Agency for Regional Health Services (AGE.NA.S.) [8], the costs of this judicial “war machine” amount to nearly 1% of Italian *gross domestic product* (GDP), about 10 billion Euros a year. The ANIA, in a hearing of the Social Affairs Committee of the Italian Parliament regarding professional responsibility of government health personnel, affirmed that the number of legal complaints against doctors and medical structures has fallen, though only slightly [9].

The ANIA president affirmed that complaints about medical malpractice have been increasing in many developed countries in recent decades and therefore are not limited to Italy. However, Italy has the highest proportion of health lawsuits settled in court in Europe (more than 90%), way ahead of France (60%) and Germany (40%), and is accordingly in last place concerning health lawsuits settled out of court. The Italian judiciary has thus become the greatest defender of the health of patients against medical malpractice [10]. Data for 2007 [6] published by the TDM highlighted a 1.8% fall in complaints against health structures, from 16,424 in 2006 to 16,128 in 2007, and a 12% rise in complaints against individual doctors, from 11,959 to 13,415.

The data published in the 14^th^ TDM report, entitled “*Diritti al taglio*” (Right to Cuts) of 2011, show that alleged medical malpractice accounted for 18% of citizens’ grievances in 2009 and 18.5% in 2010, respectively. The number of alleged medical errors fell from 63% of the total in 2009 to 58.9% in 2010, but they increased to 62.7% in 2011, while cases of “lack of attention” by health personnel (both doctors and nurses) rose from 5.8% in 2009 to 12.9% in 2010, decreasing in 2011 to 12.1%. The phrase “lack of attention” here indicates a degree of negligence that does not cause harm, but can entail incongruous and potentially harmful procedures.

In the cited report, alleged malpractice accounted for 17.7% of all health problems reported by citizens in 2012. Compared to the 2007–2011 reports, the above report found a considerable increase in medical legal issues and litigation related to therapeutic errors, especially in certain branches of medicine such as Orthopaedics and Traumatology (accounting for 32.1% of cases), General Surgery (11.2%), Ophthalmology (8.2%), Obstetrics and Gynaecology (7.8%), Neurology (6.5%), Dentistry (5.3%), Oncology (5.1%) and Cardiovascular Disease Management (4.3%). Concerning diagnostic errors, in first place there is Oncology (27.3%), followed by Orthopaedics (14.3%), Gynaecology and Obstetrics (9.1%), Gastroenterology (7.8%), Neurology (7.7%), Cardiology (6.7%) and Pulmonary diseases (6.3%).

Generally speaking, the main causes of the rising level of complaints about medical malpractice are: the current greater awareness of treatment on the part of patients, a considerable increase in the levels of compensation awarded by the courts, and a greater readiness on the part of the public to go to law. Indeed, the cited studies demonstrate that Italian patients have become more aware of their right to health when receiving treatment and their expectations regarding public health structures have risen, perhaps up to intolerable and often unjustified levels. A medical treatment that does not produce the desired clinical result is often interpreted by the patient as an error, whereas, in reality, it may simply be scientifically unachievable.

An increasing number of patients now use internet to search for diagnoses and treatments and then go to their family doctor or a specialist to confirm their findings. According to a study conducted in 2010 by “La Sapienza” University of Rome [11] on behalf of the Italian Ministry of Health, 6 patients out of 10 consider internet as a substitute for the family doctor. The study, conducted by an online questionnaire, was based on a sample of 2,300 people, out of which 63% were women. 58% of those questioned said they consulted first internet and only later their family doctor. Persons over 65years old accounted for a negligible part of the sample. 66% were university graduates. Patients conduct searches in order to find out about their condition, available treatments and their side effects, and to obtain information on the hospitals and their doctors. The social status of internet users is generally high. According to another study [12] published in the *Observa* periodical in 2011, one Italian out of five between 16-74 years old uses internet to search for medical advice, but most of them (60%) find the information to be unreliable. This demonstrates that the doctor-patient relationship is in crisis, with less time spent on communication.

A key role in the increasing propensity of patients to see themselves as victims of malpractice is also played by the media, which frequently take up extreme cases reported by newspapers or TV news and seek to portray them as representative of normal practice in Italian hospitals. A few years ago a national newspaper published a front-page article stating that in Italy giving birth had become dangerous, basing its claim on individual reported cases of poor health management resulting in the death of newborn children. Following this claim, a study was conducted [13] regarding the child mortality rate in Italy. The rate was found to have been 63 children per 1,000 births in 1950, 30 children per 1,000 births in 1970, and 3.3 children per 1,000 births in 2008. Thus, together with France, Italy is the safest place in the world to give birth. It took just four cases of infant mortality to set off a major scandal and demonise a health system which was basically still healthy and efficient. In such circumstances the journalists sought merely to sensationalise the news without seeking to explain the real statistics. This irresponsible form of journalism is repeated with practically every single legal case of medical malpractice.

The consequence of this is that doctors increasingly rely on defensive medicine, i.e. allowing their diagnostic and treatment strategies to be conditioned by “judicial caution” rather than their scientific convictions, with serious economic impacts arising from excessive provision of care and the over-prescription of tests, drugs and admissions. According to recent estimates by the University of Verona [14], reported in October 2014, the cost of defensive medicine in Italy now accounts for 10%, equivalent to about 13 euro billion, of overall health care spending.

A very simple definition of Defensive Medicine is not doing what one believes to be most logical from a scientific or clinical point of view, but what best shields the doctor from any attempt to be sued by the patient. Two recent studies conducted in Italy [15,16] have revealed that the greatest reasons for doctors relying on defensive medicine are the fear of being sued for medical malpractice (80.4% of respondents according to the study by Forti of the “Federico Stella” Centre and 69% according to the study by Catino and Locatelli) and the fear of receiving a demand for compensation (59.8% of respondents according to Forti and 50.4% according to Catino and Locatelli).

As well as the doctor's fear of being taken to court by the patient, another reason for defensive medicine is the poor organisation of complex health structures, in which the protocols of reference do not adequately specify roles and responsibilities or accepted norms of behaviour such as diagnostic and therapeutic procedures, or standards to be adopted in case of emergency/urgency. The weight of any court case thus falls on the individual doctor, who is the last link in the health system's chain of organisation. Doctors consequently end up having to deal with problems that are bigger than they are, and resort to defensive medicine, which is really just an attempt to share the weight of responsibility with others.

Other factors contributing to the growth of defensive medicine described in recent studies [17,18] include: a) the influence of previous experiences of lawsuits affecting colleagues (cited by 48.4% of respondents in a study by the Rome Provincial Order of Doctors [19] and 65.7% in the analysis by the “Federico Stella” Centre); b) the influence of previous personal experiences of lawsuits (51.8% of respondents in the study by the “Federico Stella” Centre and 34.3% in the study by Catino and Locatelli); c) the fear of the impact on one's personal reputation (16.45% of respondents in the study by the Rome Provincial Order of Doctors, 43.5% in the study by the “Federico Stella” Centre and 26.4% in the study by Catino and Locatelli). Other reasons that should not be neglected are linked to organisational aspects of the health system. In a sample of doctors from all Italian regions interviewed as part of the study by Catino and Locatelli, 71% attribute the cause of lawsuits to excessive patient numbers; 75.4% to the lack of beds; 50.2% to fatigue experienced by doctors obliged to intensive work-shifts; and 32.1% to the lack of standardised professional procedures.

Defensive Medicine is now rooted in all advanced societies; socially complex, characterised by economic wellbeing but also by significant educational and cultural imbalances among different sections of the population.

Another consequence of the rise in litigation over claims of medical malpractice is the rise in the cost of insurance premiums. The insurance premium for doctors in 2012 was 23.86% higher – about 1,000 euros more than in 2011. Claims for damages are geographically distributed as follows: 56.54% in the North and 38.57% in the Centre of Italy. Paradoxically, insurance costs per doctor are 3,828 euros in the country's top level health structures and 3,263 euros in university clinics [20].

A recent proposal by the doctors’ section of the Italian General Confederation of Labour trade union (CGIL) [17] seeks to make professional culpability a matter of civil – rather than criminal – law. This would entail an increase in insurance premiums, whose cost would not be borne by the individual doctor but by the health structure where the doctor works. These insurance policies would encourage risk management, adequately supported by the health service, in order to reduce malpractice and thus keeping insurance premiums to a minimum.

Risk management takes account of all the various complex activities undertaken to improve the quality of health treatment and guarantee the safety of the patient. Only proper risk management, which entails learning the lessons from any made mistakes, can lead to substantial changes in clinical practice, making it more adaptable to the needs of both patients and healthcare workers [18].

The Italian Government does not appear to have a strategy for resolving this serious problem, which has considerable economic and ethical-deontological consequences.

When working on diagnosis and treatment, doctors in Italy find themselves caught between the need to provide care and the potential criminal responsibility inherent in every medical act. This state of affairs obliges doctors to go to extreme lengths to prevent any professional risk, in order to protect themselves from a medical and legal point of view.

Another consequence of the increased frequency of legal proceedings against doctors is a 600% increase in the price of professional responsibility insurance premiums for surgeons over the last few decades [21]. The ANIA has finally communicated [7] data regarding health insurance policies covering the professional responsibility of health personnel: in 2011, Italian insurance companies raised about 525 million euros for health liability policies, 57% of which was accounted for by policies taken out by health government structures and the remaining 43% by policies taken out by individual doctors. The President of ANIA pointed out that the above statistics do not include premiums collected by companies based in other European countries operating in Italy under the freedom to provide insurance services, some of which are particularly active in the health care insurance sector.

Compared to 2010, payouts unfortunately increased by 5.5%. This was the main cause of the increase in the cost of premiums for professionals, probably due to a review of insurance prices made necessary by the continuing economic imbalances in the health care industry. Albeit to a lesser extent (+3.6% compared to 2010), premiums for health care structures also increased. The annual rate of premium income growth in the period 2001-2011 was 7.3% (5.5% for health care facilities and 10.3% for doctors).

### Discussion

The boom in complaints against doctors and local health authorities has had severe consequences for the Italian national health service, due to the higher direct costs in terms of legal damages paid to patients and the greater cost of insurance premiums. In addition, it has strained the relationship between doctor and patient and led to higher indirect costs arising from the adoption of so-called “defensive medicine”, which weighs heavily on health budgets.

Due to rapid technological and scientific progress, death is no longer perceived as a possible outcome of illness, but as an avoidable complication. Thus the doctor who managed the clinical case must have made an error and must pay, even in those cases where life and death are separated by a hair's breadth, regardless of human understanding and the scientific explanation of the condition. Italian hospitals have now become “Health Companies”, in which citizens’ health is a product or rather a commodity, purchased with reference to Diagnosis-Related Groups (DRG), with payment and/or reimbursement by the state for hospital services. Making health a commodity means annulling or simply losing the principle that life is sacred, which, from the theological and religious point of view, is the ultimate foundation of existence. The commoditisation of health has intensified the logical and materialistic attitude by which human beings are reduced to a purchasable item, a form of consumer goods and a source of profit – a not seen development since the abolition of slavery.

The reverence with which the figure of the doctor was treated in the past has become a curse, given a culture in which not only death is unacceptable, but people refuse to learn from it. No death occurs without an explicit accusation or an implicit suspicion concerning the doctor, in whom unlimited and acritical trust – practically the presumption of omnipotence – is placed. Medicine however is clearly not omnipotent and, just like any experimental science, is inexact and can only proceed via trial and error. In this regard we quote an aphorism attributed to the ancient Greek doctor and philosopher Hippocrates of Kos which skilfully summarises the difficulties of contemporary medicine “*Life is short, [the] art long, opportunity fleeting, experiment treacherous, judgement difficult”* [22].

Defensive medicine is related to the crisis in the doctor-patient relationship, which has a long history and is primarily based on a reciprocity of understanding and fiduciary behaviour. To a lesser extent it is based on the ethically significant value of respect for each individual patient, as well as on the professional and ethical role of the doctor, who must maintain his or her professional and intellectual autonomy [15]. It is highly damaging to the profession the relationship with patients to be structured more on legal and contractual principles than on trust. Clinical behaviours follow a logic of de-empowerment rather than the affirmation of the principles of science and conscience. The result is a betrayal of medicine’s real purpose, since doctors are now induced to behave opportunistically rather than keep faith in the fundamental principles of ethical duty and conduct , starting from the principle of beneficence [23].

As long ago as December 2001, the Italian National Bioethics Committee (NBC), in an opinion on the “Purpose, limits and risks of medicine” [24], had shown that the failures of medicine are often the most visible aspects of medical practice in the wider sectors of population, and generate collective reactions, expressed and amplified by the news media, with judicial consequences as well as claims for individual damages.

The solution to these problems lies firstly and foremost in educating the public. The involvement of society requires ethical communication aimed at all citizens, designed to inform them about nature, possibilities, limits and risks of modern medicine, in both scientific and practical terms. Communicating in an appropriate manner means providing transparent information and news even when this may be unpleasant or disappointing. Only in a context of genuine transparency it is possible, according to the NBC, to find solutions to the legal and bioethical issues surrounding medical responsibility, whose social relevance is greater than ever [25].

Media should consider, therefore, if they wish to correctly inform citizens, that in terms of health the various geographical, technological and organisational contexts are not all the same, and they should not delude citizens by emphasising results that cannot be obtained in every single health structure, both central or peripheral, in every case.

Another aspect of growing relevance in this regard is the desire to make money out of lawsuits, in a context where ethical values are continuously questioned and revised and subordinated to economic interests. The publication of reports on medical malpractice enhances this tendency by encouraging the general population to talk about this phenomenon without grasping its complexity. All this has led to the passing of increasingly specific laws that are hard to interpret and apply on various levels and in various corners of the Italian health system. The new regulations governing the provision of health services have made public medicine more bureaucratic, with the compilation of patient consent forms that are increasingly dissuasive and complex. Protocols now envisage excessive tests for the patient, in order to protect medical practitioners from being accused of not being thorough enough. This obviously drives up costs and makes life difficult for the patient, who has to undergo excessive diagnostic and therapeutical procedures [26].

Lastly, we should consider the way in which health stories are presented by the media, both newspapers and television, which seek to “sensationalise” and exaggerate any unfortunate event associated with medical or surgical procedures, without however following the story as it progresses to the legal stage, which in most cases results in the exoneration of the involved health workers.

The excessive use of diagnostic exams and medical treatments is a phenomenon which is becoming increasingly widespread in Italy and in other Western countries as a way to shield doctors against malpractice suits [27,28]. It has been shown [29] that multiple exams or excessive treatments are not clinically effective nor do they provide substantial benefit to the patient, though they may protect the physician in the case of legal conflict. Recently [30,31], various publications have begun to provide doctors with ways to avoid excessive medical treatment. In 2010 [32], the ABIM (American Board of Internal Medicine) Foundation indicated five medical procedures which were at high risk of unsuitability, as part of its “Choosing Wisely” campaign. As part of this campaign, some participants created a list, “Things Physicians and Patients Should Question”, providing specific, evidence-based recommendations which doctors and patients should discuss in order to choose the most appropriate treatment for the individual. In December 2012, in parallel with the American Choosing Wisely initiative, Slow Medicine launched the “Doing more does not mean doing better” project in Italy [33]. This seeks to reduce waste in health service provision and improve doctor-patient communication by working together with the patient to create the best individual treatment plan, in the belief that the over-prescription of diagnostic tests is not a solution to the problem of malpractice complaints. From the patient’s point of view, the reasons for over-prescribing treatments include the desire to understand the state of his/her health and the illusion that treatment is always better than non-treatment. From the doctor’s point of view they include ethical concerns over not requesting the necessary diagnostic exams, habits, fear of misdiagnosis or under-diagnosis, the desire to demonstrate his or her expertise, and the widely-held view that it is easier and faster to prescribe extra exams than to explain that they are not necessary.

Doctors today often find themselves caught among the latest pharmaceutical research, the reduced budget of the country’s health service, and the demands of patients for more and more diagnostic exams and treatments. Wen and Kosowsky [34] describe the defensive logic which has changed doctors’ behaviour in treating medical conditions. Doctors no longer consider their patients’ complete psychosocial history but immediately prescribe exams and treatments to reduce their legal responsibility in case they are accused of inexperience, imprudence or negligence.

Fundamental for the Italian health service over the next few years are the following three points: a) the reduction of national health spending must not impair the quality of care; b) there must be economic and organisational fairness among the various regions of Italy, safeguarding the weakest ones such as those in the South of the Country that find difficult to provide quality health services; c) the performance of health structures must be assessed by monitoring the quality of the service, with a view to improving the National health system.

## Conclusions

In the face of the growing number of complaints and lawsuits by patients regarding alleged errors, a two-pronged strategy is called for. The first step is to intensify health risk monitoring systems (risk management). This will not be easy in Italy due to the need to change deep-seated attitudes and the country's current economic difficulties that make it even less likely. Another aspect that needs to be looked at in greater detail in Italy is the role of legislation, which must be able to guarantee uniform regulation of the system, decriminalising medical errors and ensuring that they can be efficiently dealt with as a matter of civil law. Legal controversies in the field of health would thus be resolved in the civil courts, with reference to a specially drawn-up set of norms for regulating errors by doctors and/or health structures, avoiding interference and lengthy legal proceedings. Promoting a culture of safety in hospitals also means highlighting medical errors as much as possible, intensifying the quality of the care, without witch-hunts in the search for culprits but seeking to publicise the positive examples of good healthcare as much as possible. The health system thus needs to change from a punitive system to one that provides incentives to those who identify structural errors. This will encourage the spontaneous cessation of mistaken behaviours and improve the efficiency of diagnosis and treatment. All this will guarantee protection of doctor and patient alike, ensuring the best all-round care for citizens who need it.

Also crucial here is the role of the doctor-patient relationship, at the heart of which is communication. Information needs to flow between doctor and patient in both directions, since patients who feel that their doctor listens to them and provides adequate care will see that they are being given the best possible service. They will see the doctor as the protector of their health and will feel they can participate in choiches being made. In this way, should something unexpected happen, they will be better equipped to distinguish between an error and a genuinely unforeseeable event and will be more disposed to accept the latter for what it is.

Lastly, we need to take a look at the anomalous behaviour of the Italian mass media, who report every item of health news as a glaring example of a sick health system, subjecting health workers to trial by television before to the suitable courts.
